# Calcium imaging: A versatile tool to examine Huntington’s disease mechanisms and progression

**DOI:** 10.3389/fnins.2022.1040113

**Published:** 2022-11-03

**Authors:** Joshua Barry, Allison Peng, Michael S. Levine, Carlos Cepeda

**Affiliations:** Intellectual and Developmental Disabilities Research Center (IDDRC), Semel Institute for Neuroscience and Human Behavior, Jane and Terry Semel Institute for Neuroscience and Human Behavior, David Geffen School of Medicine, University of California, Los Angeles, Los Angeles, CA, United States

**Keywords:** Huntington’s disease, miniscope, fiber photometry, calcium imaging, two-photon microscopy (2-PM)

## Abstract

Huntington’s disease (HD) is a fatal, hereditary neurodegenerative disorder that causes chorea, cognitive deficits, and psychiatric symptoms. It is characterized by accumulation of mutant Htt protein, which primarily impacts striatal medium-sized spiny neurons (MSNs), as well as cortical pyramidal neurons (CPNs), causing synapse loss and eventually cell death. Perturbed Ca^2+^ homeostasis is believed to play a major role in HD, as altered Ca^2+^ homeostasis often precedes striatal dysfunction and manifestation of HD symptoms. In addition, dysregulation of Ca^2+^ can cause morphological and functional changes in MSNs and CPNs. Therefore, Ca^2+^ imaging techniques have the potential of visualizing changes in Ca^2+^ dynamics and neuronal activity in HD animal models. This minireview focuses on studies using diverse Ca^2+^ imaging techniques, including two-photon microscopy, fiber photometry, and miniscopes, in combination of Ca^2+^ indicators to monitor activity of neurons in HD models as the disease progresses. We then discuss the future applications of Ca^2+^ imaging to visualize disease mechanisms and alterations associated with HD, as well as studies showing how, as a proof-of-concept, Ca^2+^imaging using miniscopes in freely-behaving animals can help elucidate the differential role of direct and indirect pathway MSNs in HD symptoms.

## Introduction

Huntington’s Disease (HD) is a dominantly inherited neurodegenerative disorder that causes chorea, cognitive deficits, and psychiatric symptoms (Bates et al., 2015). HD is caused by an expansion of CAG triplet repeats (encoding glutamine) in the *huntingtin* gene (The Huntington’s Disease Collaborative Research Group, 1993; Walker, 2007). While the huntingtin protein (Htt) is typically expressed throughout the brain and body and is thought to play a role in gene expression and signal transduction, the mutant form (mHtt) preferentially targets striatal medium-sized spiny neurons (MSNs) and cortical pyramidal neurons (CPNs). At the cellular level, mHtt causes the HD phenotype through a toxic gain-of-function, leading to synapse and spine loss, cortical hyperexcitability, and striatal and cortical cell degeneration (Vonsattel and Difiglia, 1998; Tobin and Signer, 2000; Waldvogel et al., 2015).

Mouse models are widely used for investigation of HD pathogenesis. R6/2 and R6/1 are transgenic models that express a fragment of mHtt, leading to a rapidly progressing phenotype that develops symptoms as early as 4–5 weeks, and is considered a good model of juvenile HD (Li et al., 2005; Cepeda et al., 2010). Bacterial artificial chromosome (BAC) and yeast artificial chromosome (YAC) model mice, such as BACHD and YAC128, are transgenic models containing the full-length mutant *Htt* gene, often displaying neuropathology that is highly consistent with human HD (Slow et al., 2003; Gray et al., 2008). Knock-in mice (including CAG140 and Q175), carry the CAG expansion in the endogenous mouse *Htt* gene, and thus exhibit the progressive, symptomatic phenotype that replicates most faithfully human HD (Menalled, 2005).

Studies in animal models have suggested that perturbations in Ca^2+^ homeostasis play a role in HD, as altered Ca^2+^ dynamics often precede striatal dysfunction and manifestation of HD symptoms (Bezprozvanny, 2009; Wu et al., 2016; Raymond, 2017; Mackay et al., 2018). For example, mHtt can disrupt Ca^2+^ homeostasis through association with other proteins that regulate intracellular Ca^2+^ levels. mHtt also increases *N*-methyl-D-aspartate (NMDA) receptor-mediated excitotoxicity (Zeron et al., 2002; Bezprozvanny and Hayden, 2004), causing increased extracellular Ca^2+^ to enter the cell *via* extrasynaptic NMDARs (Cepeda et al., 2001). Pioneering Ca^2+^ imaging studies demonstrated that mHtt binds to the inositol 1,4,5-trisphosphate (InsP3) receptor 1, making it more sensitive to InsP3 upon its release when glutamate binds to its receptors. InsP3R1, when activated by InsP3, causes Ca^2+^ release from the endoplasmic reticulum (Bezprozvanny and Hayden, 2004; Wu et al., 2016; Czeredys, 2020), and low endoplasmic reticulum Ca^2+^ levels activate neuronal store-operated channels (SOCs), probably as a compensatory mechanism. SOCs are membrane-bound Ca^2+^-conducting channels that activate in response to depletion of Ca^2+^ stores (Putney, 1986). Because Ca^2+^ signaling is fundamentally disturbed early on in HD progression and potentially leads to development of disease symptoms, Ca^2+^ imaging techniques have the potential to visualize changes in neuronal activity associated with HD in animal models. They also provide a tool to test various hypotheses about HD pathogenesis, and visualize how intracellular Ca^2+^ dynamics differs in mouse models.

The focus of this minireview is not to describe in detail diverse Ca^2+^ imaging techniques as excellent, comprehensive reviews are already available (Werner et al., 2019; Broussard and Petreanu, 2021; Wang et al., 2021; Grienberger et al., 2022; Kim and Schnitzer, 2022; Lake and Higley, 2022). Instead, we discuss studies using some of these techniques to monitor the activity of neurons, astrocytes, and mitochondria in HD models (Table 1). Compared to imaging studies in other disease conditions, use of Ca^2+^ imaging to examine HD progression has taken longer to implement. This is partly due to the overwhelming reliance on electrophysiological recording techniques and also to the slow development of genetic mouse models of HD, associated to the fact that some models display a very severe phenotype, e.g., R6/2, and others show only very mild, protracted symptoms (BACHD, Q175). In the last section, we discuss future applications of Ca^2+^ imaging to visualize HD disease mechanisms, as well as some of our own studies showing how, as a proof-of-concept, Ca^2+^ imaging using miniscopes in freely-behaving animals can help elucidate the role of CPNs and direct and indirect pathway MSNs in HD symptoms. We apologize for any omissions of the already vast literature on Ca^2+^ imaging in HD.

**TABLE 1:** Ca^2+^ imaging methods applied to the study of Huntington’s disease.

Imaging technique	Type of preparation	Spatial resolution	Equipment cost	Tissue damage	HD papers published
Confocal or 2-PLSM	Cell cultures or slices	Individual cells, organelles	$$$	Tissue no longer intact in *in vitro* systems of dissociated cells or brain slices	Fernandes et al., 2007; Lim et al., 2008; Wang et al., 2013; Jiang et al., 2016; Fernandez-Garcia et al., 2020; Oikonomou et al., 2021
	Head-fixed (freely moving limbs)	Individual neurons (depth of ∼1 mm)	$$$	Inflammation; potential damage to dura and cortical surface during craniotomy and cranial window implantation	Burgold et al., 2019; Donzis et al., 2020
Fiber photometry	Freely-moving	Population signal (depth ∼2 mm)	$	Inflammation; tissue displacement by optic fiber implantation (200 μm diameter fiber)	Koch et al., 2022
Miniscope	Freely-moving	Individual neurons (depth ∼150 μm from GRIN lens)	$–$$	Inflammation; tissue displacement due to GRIN lens implantation (0.5–1.0 mm diameter lens)	No papers yet Proof-of-concept

### Fluorescence confocal and 2-photon laser scanning microscopy

Past investigations into circuits and pathways involved in HD development have been limited by difficulty recording from large neuron populations and constraints in temporal and spatial resolution. While a leading tool for investigating neuronal dynamics has been electrophysiological recordings of neuron electrical activity in the form of action potential firing (both extracellularly and intracellularly) due to high temporal resolution, these methods can be limited in spatial resolution, specificity of neuron types, or are typically able to record from only a few neurons. This presents challenges in investigating altered circuits in HD due to the relatively isolated neuronal recordings.

**FIGURE 1:**
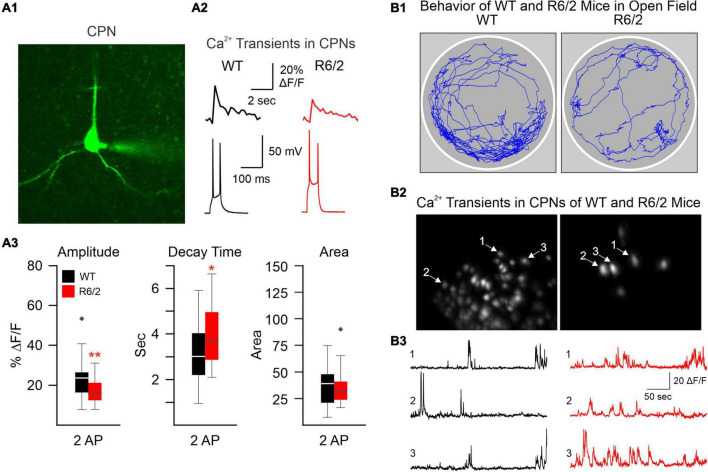
**(A)** We used two-photon microscopy to examine Ca^2+^ influx induced by action potentials (APs) in layer II cortical pyramidal neurons (CPNs) from R6/2 model mice. **(A1)** A CPN was patched and filled with a Ca^2+^ indicator (OGB-1). **(A2)** Action potentials (APs) evoked by 50 ms depolarizing current steps from the resting membrane potential. Accompanying Ca^2+^ transients are shown above the APs. **(A3)** The amplitude of somatic Ca^2+^ transients was reduced in R6/2 mice compared to controls. This reduction was compensated by increased decay times, which could lead to reduced Ca^2+^ buffering capacity [modified from Oikonomou et al. (2021)]. **(B)** A miniscope and a video camera above a cylindrical arena were used to visualize Ca^2+^ transient activity and behavior of WT and R6/2 mice. **(B1)** Movement tracking during 5 min behavior video recording. Notice the sparsity of movement in the symptomatic R6/2 mouse. **(B2)** Images of fluorescent CPNs in layer V of the same WT and symptomatic R6/2 mice. **(B3)** Ca^2+^ transients from three selected CPNs shown in **B2**. *p < 0.05, **p < 0.01, and black diamonds are outliers for the box and whisker plots.

The development of confocal and two-photon laser scanning microscopy (2-PLSM) has opened the door to neuron recordings in deeper tissue, using laser excitation methods that are able to reduce background fluorescence noise and discern individual neurons (Svoboda and Yasuda, 2006). Pioneering groups developed 2-PLSM and recognized its potential to solve problems with photobleaching, depth, and spatial resolution present in other imaging methods, such as confocal and light microscopy (Denk et al., 1996; Svoboda et al., 1997). When utilized for Ca^2+^ imaging, fluorescent genetically encoded Ca^2+^ indicators (GECIs) bind to intracellular Ca^2+^ and emit fluorescent signals upon excitation, which can be acquired through the microscope. Since Ca^2+^ influx is associated with action potentials, it provides an indirect indication of neuronal firing activity. Cell type-specific expression of the Ca^2+^ indicator can be used to limit fluorescence to neurons of interest. In brain slices, 2-PLSM allows for recording from 50 to 100 cells simultaneously and extracting spike estimates from Ca^2+^ signals (Deneux et al., 2016), opening the possibility to compare neuronal Ca^2+^ activity in HD and wildtype (WT) mice. Fernandez-Garcia et al., for example, used fluorescence imaging of striatal and cortical cell cultures from R6/1 and WT mice to examine Ca^2+^ dynamics. Notably, spontaneously active neurons and coordinated ensemble activity were reduced in striatal but not in cortical cultures, suggesting reduced ability to integrate excitatory inputs (Fernandez-Garcia et al., 2020). However, altered Ca^2+^ influx may occur in the cerebral cortex of HD model mice. We used 2-PLSM to examine, in brain slices, Ca^2+^ influx associated with action potentials evoked by depolarizing current pulses in CPNs of R6/2 mice. We found reduced amplitude of somatic Ca^2+^ transients in R6/2 mice compared with controls (Figure 1A). This reduction appeared compensated by increased decay times, which could be deleterious due to reduced Ca^2+^ buffering capacity in HD neurons (Oikonomou et al., 2021).

Reduced Ca^2+^ buffering capacity may be associated with mitochondrial dysfunction. Indeed, mitochondrial Ca^2+^ levels also are dysregulated in HD. Using Ca^2+^ probes that do not permeate the mitochondrial membrane as indicators of extramitochondrial Ca^2+^, it is possible to indirectly monitor influxes of Ca^2+^ into the mitochondria, as well as total Ca^2+^ retained in the mitochondria upon opening of permeability transition pore (PTP) and Ca^2+^ release into the cytosol (Ferreira et al., 2018). Additionally, small molecule fluorescent Ca^2+^ indicators (e.g., Fura-2, Fura-FF) and GECIs (e.g., GCaMP, Cameleon, Pericam) can be made to specifically target organelles such as the mitochondria, directly providing insights into mitochondrial Ca^2+^ transport (De Nadai et al., 2021). Many of these experiments are done on neural cell cultures, though mitochondrial Ca^2+^ imaging can also be used on isolated brain mitochondria, with potential to be used *in vivo* with multiphoton microscopy (Calvo-Rodriguez et al., 2021). Using diverse methods to visualize mitochondrial Ca^2+^ dynamics, it was found that increased mitochondrial Ca^2+^ uptake promoted the oxidative stress and accumulation of mitochondrial DNA mutations in striatal neurons associated with HD (Wang et al., 2013). Indeed, the authors observed more mitochondrial Ca^2+^ loading in YAC128 compared to WT cells, and suggested reducing mitochondrial Ca^2+^ uptake as a potential therapeutic target for HD. In a similar study, it was reported that mitochondria in YAC transgenic mice are more sensitive to Ca^2+^-induced activation of PTP, leading to enhanced NMDA-induced apoptosis (Fernandes et al., 2007). Enhanced sensitivity of PTP in HD knock-in mouse models also has been observed (Lim et al., 2008). The contribution of reactive astrocytes to cell death in HD, and the changes in Ca^2+^ activity in HD model mice, also indicate the potential utilization of Ca^2+^ imaging on astrocytes as a potential investigative topic. Notably, even before overt astrogliosis, striatal astrocytes from symptomatic R6/2 mice display reduced spontaneous Ca^2+^ signals compared to astrocytes from WT animals (Jiang et al., 2016).

When recording in cell cultures or brain slices it is difficult to ascertain more complete neural circuits as they function in living mice. Fortunately, 2-PLSM imaging can also be utilized on head-restrained, behaving mice. This is done by performing a craniotomy to expose the area of interest, placing a glass cover over the cranial window, and recording through a 2-PLSM lens placed above the window (Donzis et al., 2018), which can record depths up to 1 mm. During recording, the head must be secured in place to avoid motion artifacts, and mouse behavior is constrained to movement on a ball treadmill. In live mice, two-photon microscopy allows for longitudinal imaging, potentially being able to visualize the differing Ca^2+^ dynamics during mouse behavior in various neuronal populations, e.g., CPNs and interneurons, as the disease progresses. Burgold et al. used 2-PLSM to monitor the activity of CPNs in layers II/III of the primary motor cortex in awake, behaving R6/2 mice and WT littermates. CPNs in HD mice showed increased activity within 1 week before the onset of motor deficits, suggesting reduced cortical inhibition (Burgold et al., 2019).

In our lab, Donzis et al. utilized 2-PLSM to examine cortical network dynamics in R6/2 and Q175 mice and evaluate changes in cortical microcircuits. They observed Ca^2+^ transients during motion and non-motion epochs and found significant alterations in behavior and cortical neuron activity in R6/2 and Q175 mouse models compared to WTs (Donzis et al., 2020). Some alterations were similar in the two HD models, such as a reduction in Ca^2+^ transient amplitude, indicative of decreased bursting activity and consistent with electrophysiological findings (Walker et al., 2008). Other alterations included changes in Ca^2+^ transient frequency during motion and non-motion epochs, with decreased frequencies in both epoch types in R6/2 mice and increased frequencies in motion epochs in Q175 mice. This indicates that neuronal activity from these populations is more significantly altered during motion in HD models. Both models also displayed decreased CPN interneuronal correlations, which suggests disrupted communication as the disease progresses (Donzis et al., 2020).

Although 2-PLSM can provide much greater context in a moving mouse than in slices, the fixation of the headbar during experiments and the limitations of movement in such experiments may alter mouse behavior and brain activity, and is thus not representative of fully free movement. For example, symptomatic R6/2 mice tend to move less in natural conditions compared to WTs. In contrast, when head-fixed and placed on a ball, they tend to move more. This can be caused by R6/2 mice being less able to balance on the ball and thus compensating by adjusting position to maintain balance (Donzis et al., 2020). Additionally, certain mouse models, such as the R6/2, are more susceptible to stress, and their altered behavior when moving on the ball could reflect the stressful environment. Advancements such as the use of an air-lifted track (Kislin et al., 2014; Nashaat et al., 2016) or linear self-paced treadmills (Muldoon et al., 2015) could potentially cause less stress and balance challenges to the head-fixed mouse than the spherical treadmill.

Many new implementations of 2-PLSM are currently in development, such as creation of a two-photon microendoscope, in which an endoscopic (GRIN or fiber-based) lens is surgically implanted into the mouse brain expanding imaging to deep tissues inaccessible by traditional two-photon benchtop microscopes, and allowing use in freely-moving animals (Jung and Schnitzer, 2003; Kucikas et al., 2021). Challenges of endoscopic systems lie in minimizing tissue damage when implanting into the brain, which is comparatively higher than recording through a cranial window as GRIN lenses cause damage to tissue around the implant site (Meng et al., 2019; Zhang et al., 2019; Kucikas et al., 2021).

### Fiber photometry

Fiber photometry is another way to measure bulk neuronal activity, using a fiber optic cannula probe implanted into the brain carrying pulses to excite Ca^2+^ indicators, whose fluorescence is then carried back through the fibers (Li et al., 2019). It provides a way to record neuronal activity in freely-moving animals due to the flexibility of the fiber optic cable. The small size of the optic fibers (∼200 μm) also reduces damage to surrounding brain regions (a disadvantage in other Ca^2+^ imaging modalities), and allows for recordings from multiple brain regions simultaneously, as well as in deep tissue (Girven and Sparta, 2017; Wang et al., 2021). These recordings can then be correlated with animal behavior (Martianova et al., 2019). The stability of fiber photometry (when implanted in the brain) also allows for longitudinal recordings, correlating changes in Ca^2+^ dynamics as HD progresses. The popularity of fiber photometry has been promoted by groups such as the Deisseroth’s Lab, who have used this technique to record and reveal real-time, behavior-related circuits (Gunaydin et al., 2014; Kim et al., 2016).

Fiber photometry has been used to record Ca^2+^ fluorescence from the striatum of YAC128 model mice while undergoing a series of motor learning and coordination tasks, specifically the rotarod test (Koch et al., 2022). The authors investigated signaling changes by studying Ca^2+^ dynamics at different stages of the disease and found that even pre-manifest YAC128 mice display altered striatal activity associated with rotarod performance compared to WT. These alterations include weaker inverse correlation between latency to fall and striatal activity and different patterns of brain activity accompanying paw slip events, suggesting decreased synaptic plasticity associated with motor learning.

However, a major drawback of fiber photometry is its inability to record from individual cells. Instead, fiber photometry can record from populations of neurons (defined genetically to express GECIs), which has much lower spatial resolution and does not allow for visualization of specific neuronal interactions. It has also been observed that fiber photometry is more correlated with non-somatic Ca^2+^, as opposed to somatic Ca^2+^, and thus it is not a one-to-one representation of spiking activity (Legaria et al., 2022). Despite these limitations, fiber photometry has the potential to be used in conjunction with other techniques, such as *in vivo* electrophysiology (Patel et al., 2020), allowing for Ca^2+^ transients from specific subpopulations of neurons to be combined with measurements of electrophysiological activity and optogenetics. This would allow for *in vivo* recordings of Ca^2+^ dynamics in specific circuits, correlated with how firing patterns change with different behaviors or disease progression. Its simplicity, versatility, and low entry barrier are major draws to fiber photometry. Therefore, although fiber photometry has been used sparingly to investigate changes associated with HD, the many recent developments make it a promising tool.

Current areas of development for improving fiber photometry include, aside from simultaneous use with other imaging modalities, improving multi-fiber photometry, which allows for increased flexibility of probe placement and higher density channel placement (Sych et al., 2019), improving visualization of dynamics in various circuits (such as striatal direct/indirect pathways for HD), as well as optic cannulas with thinner tips to allow for more flexible placement. As techniques to separate signals continue to improve, simultaneous visualization of two neuronal populations using Ca^2+^ indicators of different colors (Meng et al., 2018) could potentially detect changes in activity of striatal direct and indirect pathway neurons associated with HD symptoms.

### Miniscopes for use in Huntington’s disease mouse models: Proof-of-concept

Miniscopes represent another method that builds upon confocal microscopy and utilizes miniature head-mounted fluorescence microscopes to visualize Ca^2+^ transients in the brain *via* GECIs. Similar to other Ca^2+^ imaging methods, neuron cell types selected by genetic expression of GECI-GFP protein can be visualized using the miniscope. Miniscopes use GRIN lenses in the brain, allowing for visualization of individual fluorescent cells expressing GECI-GFP (Zhang et al., 2019). Pioneered by the Schnitzer group, the lightweight, stable miniscope allows for neuronal activity to be recorded with minimal motion artifacts from a freely-moving mouse with unrestricted movement, time-correlated with behaviors, providing an advantage over traditional two-photon microscopy methods (Ziv and Ghosh, 2015). Additionally, experiments comparing miniscopes with stationary 2-PLSM show that there is little variability in signal amplitude and signal-to-noise ratio between the two methods (Glas et al., 2019), and miniscopes are a highly reliable method for neuronal activity recordings.

Currently, our lab is using the UCLA miniscope (v3) to record Ca^2+^ transients in neurons from layers V to VI in the cortex to image CPNs. Similar to the reductions in amplitude observed in our previous publication using 2-PLSM (Donzis et al., 2020), we found reduced amplitude of Ca^2+^ transients in symptomatic R6/2 mice (Figure 1B). However, in contrast to our previous study in head-fixed animals, we also found that R6/2 mice moved much less than WT mice, demonstrating that Ca^2+^ imaging using miniscopes better reflects the HD phenotype of symptomatic R6/2 mice, namely hypokinesia. In addition, using Cre-expressing mice in different neuronal populations we have been able to visualize specific types of interneurons (in PV- and SOM-Cre mice) as well as direct and indirect pathway MSNs (in D1- and A2A-Cre mice).

Miniscopes represent an improvement on challenges faced by other forms of Ca^2+^ imaging regarding low spatial resolution and limitations on behavior and movement. The remaining limitations with miniscopes, but also present with other imaging techniques, include general obstacles with Ca^2+^ imaging regarding the duration of the transient signal. Ca^2+^ imaging is in general slower to respond than voltage-based imaging due to delay in binding of Ca^2+^ indicators. Also, due to slow release of Ca^2+^ indicators, cell fluorescence can remain after the firing of action potentials. The extended decay time (in the order of seconds) of the signal limits visualization/deconvolution on the duration of Ca^2+^ responses, and only reliably represents the beginning of the signal. For some proposed mechanisms for disease pathology of HD (Raymond, 2017) that suggest that changes in NMDAR response duration are responsible for apoptosis, Ca^2+^ imaging can be less effective. Additionally, most miniscopes utilize single-photon Ca^2+^ imaging, which has higher background fluorescence and tissue scattering of light, which somewhat limits its spatial resolution compared to 2-PLSM. Recently, lightweight (2–3 gr) miniature 2-photon microscopes are being developed (Zong et al., 2017, 2021, 2022), applying the deep penetration and higher resolution of two-photon microscopy to a freely-moving animal.

Though still in development, these systems enable monitoring with two-photon imaging over weeks, with minimal restrictions on behavior. However, similar to other methods using implanted lenses, the GRIN lenses used in miniscope imaging can cause some brain tissue displacement or damage.

Future research with miniscopes is focused on imaging more cell types in a circuit, potentially using multiple GECIs with wavelengths that can be distinguished, thus visualizing Ca^2+^ activity from multiple neuron populations (Werner et al., 2019). Additionally, projects enabling simultaneous extracellular electrophysiology recordings (Aharoni and Hoogland, 2019) expand the current methods to investigate neural circuits. The development of pipelines such as MIN1PIPE and CaImAn with built-in motion correction and denoising features greatly improved accessibility to miniscope imaging (Lu et al., 2018; Giovannucci et al., 2019). Behavioral classification to aim for closed-loop miniscope systems are also in development to streamline data processing and allow easier extraction of animal’s behavior during recording (Aharoni and Hoogland, 2019). Current improvement of wireless miniscopes (Barbera et al., 2019) also continues to minimize behavioral interference during recording, as well as the damage produced by the GRIN lens.

## Conclusion

This minireview has presented several different Ca^2+^ imaging modalities and their applications to investigate the development and disease progression of HD in mouse models. These methods have been used to compare alterations in Ca^2+^ dynamics, which are likely key in HD manifestation. Current advances in Ca^2+^ imaging will make it possible to use Ca^2+^ imaging in conjunction with other methods to corroborate and formulate hypotheses about specific neuronal networks, dynamic alterations, and circuits involved with HD. For example, use of miniscopes in different Cre mouse lines may be able to elucidate the potentially differential role of striatal direct and indirect pathway neurons during the onset of motor symptoms, as well as the role of different types of interneurons in striatum and cerebral cortex.

## Author contributions

All authors wrote the first draft of the manuscript, worked on revision, read, and approved the submitted version of the manuscript.
